# Who decides who goes first? Taking democracy seriously in micro-allocative healthcare decisions

**DOI:** 10.1007/s11019-025-10263-w

**Published:** 2025-03-15

**Authors:** Davide Battisti, Chiara Mannelli

**Affiliations:** 1 Department of Law, University of Bergamo, Via Gianbattista Moroni, 255, 24127 Bergamo, BG Italy; 2 Istituto Superiore di Sanità, Bioethics Unit, Via Giano Della Bella, 34, 00162 Rome, Italy

**Keywords:** Allocation of healthcare resources, Democracy, Distributive justice, Formal justice, Micro-allocation, Political representative

## Abstract

The structural scarcity of healthcare resources has deeply challenged their fair distribution, prompting the need for allocation criteria. Long under the spotlight of the bioethical debate with an extraordinary peak during the recent COVID-19 pandemic, micro-allocation of healthcare has been extensively discussed in the literature with regard to issues of substantive and formal justice. This paper addresses a relatively underdiscussed question within the field of formal justice: who should define micro-allocation criteria in healthcare? To explore this issue, we first establish formal requirements that must be met for allocation criteria to be considered fair and legitimate. Then, we introduce three possible answers to the research question: the attending physician, the team of physicians, and the team of experts. We discuss and then reject all of them, arguing that the task of defining allocation criteria should be assigned to a political representative, supported by a cross-disciplinary team of experts. This proposal is based on the need to take democracy seriously as a tool for making substantive allocative decisions in light of the inevitable disagreement on such matters within a community. To support this claim, we present two key arguments—the democracy argument and the consistency argument. We also pre-emptively respond to two significant critiques: the too-specificity of the decision critique and the catastrophic outcomes critique. In conclusion, we argue that our proposal offers the fairest and most legitimate decision-making process for healthcare micro-allocation.

## Introduction

After the COVID-19 pandemic, there has been increasing attention in the bioethical debate regarding the micro-allocation of healthcare resources. This can involve prioritizing at the bedside, deciding which patient should be treated first, or—in the most dramatic situations, such as those that occurred in some countries during the pandemic—even rationing, namely deciding which patients should receive treatment and who should go without it.^1^ The debate has often focused on identifying ethically justifiable criteria for making decisions in conditions of scarcity (Emanuel et al. 2020; Craxì et al. 2020; Vinay et al. 2021; Meier 2022). Such issues are also referred to as substantive justice issues, since they embody an evaluative judgment on what constitutesthe right choice from an ethical standpoint in a given context, focusing on the content of the principles of justice. A substantive justice question in this regard is, for instance, asking whether or not we should prioritize younger patients in the Intensive Care Unit (ICU) during times of scarcity (Persad and Joffe 2021; Albertsen 2023) or whether responsibility for alcohol-related end-stage liver disease should be considered relevant when allocating organs (Hu and Primc 2023; Albertsen 2016). Nevertheless, another segment of the debate has explored issues of justice beyond those related to defining allocative criteria, concerning how substantive criteria are chosen and the requirements that they should meet within a democratic society—issues of formal justice. From this perspective, we can identify at least three main questions: (a) through what procedure and guided by what formal requirements should the allocative criteria be defined? b) When should the allocative criteria be decided? and c) who should define the allocative criteria? With regard to (a), there have been some proposals in the debate, both in general (Daniels and Sabin 1997, 2008) and related to more specific allocative contexts (Battisti and Camporesi 2023). Regarding (b), the need for ethical preparedness for scenarios of severe scarcity that require reflection on allocative criteria in advance has been highlighted (Battisti et al. 2021; Mannelli 2021; Emanuel and Persad 2023). Although discussed (Battisti and Picozzi 2022; Camporesi and Mori 2020), (c) has not received systematic analysis so far and thus lacks a comprehensive proposal in the debate. The aim of this contribution is to address this question from a normative perspective.

The paper is structured as follows. In Sect. “Formal justice requirements to ensure fair and legitimate allocative decision”, we briefly discuss the formal requirements that healthcare allocation decisions should meet in a democratic context. Establishing formal requirements will be fundamental for analyzing several possible responses to (c), which will be addressed in Sect. “Who should decide? Some possible answers”. In particular, we will examine three responses: the attending physician, the team of doctors, and the team of experts. We will reject the first two options precisely because they fail to meet the formal requirements for a fair and legitimate^2^ allocative choice. In Sect. “Taking democracy seriously in micro-allocative decisions: the role of the political representative”, we will also reject the latter proposal, by arguing in favor of the thesis that the political representative, assisted by some experts, should make the decision. This perspective arises from the need to take democracy seriously as a tool for making substantive allocative decisions in light of the disagreement on such issues within a community. In Sect. “Two possible critiques”, we preemptively respond to two criticisms of our position: the too-specificity of the decision critique and the catastrophic outcomes critique, concluding that our perspective ensures the fairest and most legitimate choice possible in the context of micro-allocation in healthcare. This aspect is particularly relevant in a world where it is increasingly urgent and necessary to address such issues in time to effectively tackle current and future situations of scarcity.

## Formal justice requirements to ensure fair and legitimate allocative decision

Before determining who should decide the allocation criteria, it is essential to first outline the formal requirements needed to ensure that the decision is both fair and legitimate. As we will see, assigning decision-making power to a specific actor can directly weaken or promote these requirements. Therefore, the selection of the decision-maker depends on her ability to comply with them.^3^ As already said in the introduction, we can find in the literature some helpful accounts. The most famous is the so-called “accountability of reasonableness” proposed by Daniels and Sabin (1997, 2008), defined as “the idea that the reasons or rationales for important limit-setting decisions should be publicly available. In addition, these reasons must be ones that ‘fair-minded’ people can agree to be relevant to pursuing appropriate patient care under necessary resource constraints” (Daniels and Sabin 1998). Offering a comprehensive analysis of this account is beyond the scope of this section; we limit ourselves to arguing that the considerations we present here can be compatible with those discussed by Daniels and Sabin.

Any decision to allocate health resources inevitably results in some people benefiting (the winners) and others not (the losers). However, as long as a national health system stands—such as the British or Italian one, which guarantees at least a certain level of health care for the entire population of the country—both winners and losers have a legitimate right to health resources. This means that both stand preliminarily in a condition of equality with respect to the claims they can make towards the national healthcare system, from which a presumption of equal distribution of health resources follows. But since we live in a context of structural scarcity of resources, it is not always possible to guarantee such equal distribution. For this reason, it becomes crucial to identify allocation criteria. How is it possible to respect the equality described above in a context of scarcity where allocation criteria are necessary? Ideally, in a democratic context, these allocation criteria must at least be justifiable and acceptable to all. This assessment stems from the concept of the social contract, according to which principles of justice should be decided by people who regard each other as equals who must reach some sort of public agreement about them (Rawls 2009).

Therefore, the differences among people imposed by allocative criteria must be justified based on general, impartial, and universally acceptable reasons. In this view, fair allocation requires, at a minimum, that it respects the *principle of* *treating like cases alike*, according to which like cases should be treated similarly and different cases differently (Aristotle 2009 [fourth century B.C.]). Moreover, the allocative decisions that set the limits of health resources and their justifications should be public and transparent for all those over whom those allocative decisions claim authority. This is what Daniels and Sabin call the *publicity condition* which applies not only to allocative decisions, but also to making explicit how such decisions were reached, and the grounds on which they were reached. This, of course, involves outlining their underlying value-judgements and ethical commitments. Furthermore, according to Daniels and Sabin, it is not enough that allocative decisions and rationales for them are public and accessible. Reasons supporting them should also meet certain conditions: they should be accepted as “relevant by fair-minded people who are disposed to finding mutually justifiable terms of cooperation” (Daniels and Sabin 2008). This requirement, called the *relevance condition*, implies a reason-giving approach involving people in an “open conversation” on the allocation of scarce resources, ensuring that everyone affected by such decisions can understand and accept the reasoning behind them.

Admittedly, the relevance condition has been deeply criticized in the literature due to the difficulty in determining which reasons can actually be considered relevant (Ford 2015; Friedman 2008; Rid 2009; Lauridsen and Lippert-Rasmussen 2009). It is beyond the scope of our paper to address these criticisms, and we limit ourselves to arguing that there is a minimum level of relevance that we can consider acceptable: reasons free from logical fallacies, based on clearly stated assumptions, which coherently lead to conclusions, and are not contradicted by empirical evidence, which are—as already mentioned—general, impartial, and universally acceptable, can be considered relevant reasons. In other words, these reasons are those that can be presented for public discussion and are not based on scientifically or philosophically disreputable views (Reichlin 2022).

As we will discuss in depth below, different allocation criteria in micro-allocation can meet these requirements; therefore, these considerations do not exhaust the discussion regarding which criterion is substantively just. Instead, these formal requirements constitute the means through which different allocation paths can be considered fair and legitimate. Hence, regardless of the substantive allocative criterion chosen, every decision regarding healthcare allocation must respect such formal constraints. This aspect is even more relevant if we expect to encounter substantial disagreement within a society regarding the substantive criteria to be employed in allocative decision-making. For example, there could be division on the principles that should guide such choices or, alternatively, even assuming a reasonable agreement on the principles, there could be discord on how they should be concretely implemented in the decision-making process in clinical practice. We come back to disagreements in allocation in Sect. “Two possible critiques”. Here, we aim only to highlight that such divisions make it crucial to ground each decision regarding allocative criteria in a procedure that ensures respect for the formal constraints mentioned above.

## Who should decide? Some possible answers

Given the preliminary theoretical framework, we can now address the question of who should make the decision concerning allocative criteria in healthcare. We analyze three possible answers—the attending physician, the team of doctors, and the team of experts.

### The attending physician

Some may argue that, particularly concerning the debate on bedside rationing (Ubel and Goold 1997), the attending physician should decide the allocative criteria. Although in the macro-allocative context the decisions regarding resource allocation do not seem to directly concern the physician, in the micro-allocative setting some might argue that they should play a crucial role. In this line, the physician might be required to act as what has been called *double* *agent* (Angell 1993): The Physician Charter on Professionalism, jointly drafted by the American Board of Internal Medicine Foundation and the European Federation of Internal Medicine, indeed states that physicians must promote the best interest of patients while ensuring that health resources are distributed equitably in society (ABIM 2004). Thus, in conditions of scarcity, the physician would be called upon to balance these conflicting obligations, evaluating their relationship with all patients, including future ones, in the same way as their relationship with the current individual patient (Brennan and Lee 2004). This means that the attending physician would be in charge of establishing the allocative criteria because such a task falls within their professional duty. The argument regarding the physician's obligations is reinforced by pragmatic considerations: the attending physician is, in fact, the person most aware of their patient’s clinical conditions and, at the same time, has knowledge of the extent of scarcity in which the hospital operates. In other terms, the physician would be the best-placed person to make choices informed by all relevant facts and circumstances.

Such a position has been criticized from several perspectives. For instance, according to some authors, leaving the decision to the attending physician would deny the priority of the individual patient’s well-being over the interests of society (Wyller 2017; Pellegrino 1994). This criticism reaffirms the traditional medical ethics “individualist” conception of the physician against the “systemic conception” of the physician allocator, which finds its justification in the dual nature of the doctor-patient relationship (Glover 2019). Notwithstanding, setting aside this class of critiques, we claim that this proposal faces, first and foremost, several formal justice problems.^4^ Considering what we argued in the previous section, the need to respect the formal requirements prevents the approach of the physician as the decision-maker from being successful. Leaving such a decision to the attending physician exposes to the risk that the doctor could decide based on their own values, without these being scrutinized in a process analyzing the reasons at play. Thus, the doctor’s choice could be subject to arbitrariness or even controversial and unjustifiable ethical positions. Moreover, these decisions could also be influenced by cognitive biases or emotional factors that make the choice of criteria extremely context-dependent (Persson et al. 2018) and potentially influenced by aspects that are not morally relevant. A noteworthy case in this regard is the weight physicians tend to give to the difference between withdrawing and withholding treatments (Chung et al. 2016; Giannini and Consonni 2006). This arbitrary approach unavoidably paves the way to inequalities within the same healthcare system or hospital. Two physicians could treat the same patient, or two patients affected by a similar condition, differently despite being in similar scarcity circumstances, falling short of the treating similar cases alike principle. Moreover, it might be more complicated for such a context- and emotion-influenced approach to meet the requirements of transparency and publicity we discussed above. To meet such requirements, allocative decisions should not be considered an exclusive prerogative of the attending physician (Battisti 2023; Battisti and Picozzi 2022), who should rather focus solely on treating their patients within the constraints of already set allocative limits.

To our argument, someone might object by reiterating that only the attending physician is aware of the clinical situation of their patients, and this determines their inevitable role in deciding how to allocate resources. This potential critique allows us to distinguish between two concepts that here seem to be confused. There is a substantial difference between choosing the criteria and implementing them in the clinical setting. The former involves identifying general criteria, as well as the reasons supporting them, that can be applied in the clinical context considering the patients’ conditions. Setting the criteria has nothing to do with specific knowledge of patients’ clinical conditions. On the contrary, when it comes to applying them, understanding and evaluating a patient's specific conditions is crucial. In this respect, there is a need to consider the context as appropriately as possible to implement the criteria. Therefore, while the former concept should not fall to the physician, the latter should, precisely in light of the position that the attending physician occupies in the clinical setting. Still, we can concede that in this scenario some degree of discretion is inevitable. Even by defining the criteria upstream through a fair procedure, this does not exclude situations in which the attending physician must make allocative decisions: however precise, criteria may fail to exhaust the emergence of every allocative dilemma that could arise in clinical practice. Nonetheless, this does not imply that all aspects of the allocative decision must remain the prerogative of the attending physician. On the contrary, there is a need to minimize as much as possible this element of discretion.

Finally, our claim is not committed to arguing that clinicians should have no say in the decision of the criteria since they are inextricably involved in such a decision. In this sense, it is possible to recognize, in line with Tilburt (2014), that tphysicians can occupy different roles depending on the circumstances. Among others, physicians can act as caregivers, administrators, public health officials, medical educators, policy makers. It follows that when the doctor acts as an attending physician, they cannot be called upon to decide how to allocate scarce resources; but this does not mean that the same doctor cannot, to some extent, be involved in defining such criteria in different circumstances and under a different role. This crucial aspect allows us to investigate the second hypothesis for our answer, namely whether a team of doctors—who are not acting as attending physicians at the time of the decision—meets the formal requirements listed above to define the allocative criteria.

### A team of doctors

A team of doctors could present some advantages compared to the previous approach. In fact, this solution would require the team members to reach an agreement onallocative criteria which should then be applied in a clinical setting. This approach might allow us to respect the principle of treating similar cases alike, leaving less room for arbitrariness. Moreover, a team of doctors would necessarily promote greater transparency in the chosen criteria.^5^ The preliminary discussion on the criteria—namely, before their implementation—and the subsequent communication of the chosen criteria to the community would at least allow community members to be informed about the selected criteria and to know in advance the decisions they might be subject to. In this scenario, the “Clinical ethics recommendations for the allocation of intensive care treatments in exceptional, resource-limited circumstances” drafted by the Italian Society of Anesthesia Analgesia Resuscitation and Intensive Care (Vergano et al. 2020) constitute a relevant case. During the first wave of the COVID-19 pandemic, this document developed by a group of Italian clinicians represented the first effort to set transparent criteria for the allocation of ICU resources, by making them public and accessible in a context of serious emergency and profound scarcity.

We argue that one of the main concerns associated with the team of doctors’ approach is twofold: first, making transparent allocative decisions is not enough to meet the condition of publicity. It is not enough to make transparent allocative decisions, because—as we argued—reasons behind them should be transparent too. Furthermore, these reasons should also meet the relevance condition, being accepted as relevant by fair-minded people who are disposed to finding mutually justifiable terms of cooperation. This means that the criteria must be supported by solid and understandable arguments where the premises are clear and explicit, and consistent with the conclusions of the deliberation. There may be some exceptions, but generally a team of doctors lacks to some extent all the expertise needed to provide a comprehensive justification of the allocative criteria. Although physicians may be particularly competent in the assessment of some factors relevant to allocative decision-making, such as diagnostic and prognostic aspects in clinical settings, the allocative choices also involve other aspects that go beyond the physician’s areas of competence.. In other words, a team of doctors alone lacks other expertise required to reach a fair and legitimate allocative decision. In light of these considerations, there are reasons to include not only physicians, but also other experts among those who should contribute to identifying the allocative criteria.

### A team of experts

To assess whether a team of experts might be an appropriate solution, we need to identify which expertise, beyond medical expertise, should be included in this team. To answer this question, it is important to remember that, despite not always being obvious, each allocative decision, along with technical considerations, implies value judgments. In light of the reasons mentioned above, these value judgments must be justifiable and deemed acceptable by everyone. We therefore believe that ethics expertise is needed in order to analyze and elucidate moral concepts or to evaluate arguments for or against criteria, to understand the assumptions and implications of different normative positions that can justify allocative criteria. By “ethics experts” we do not mean individuals who hold the “true” answer to moral questions or who know what the right thing is in an objective way. Rather, following Yoder, we argue that “[A] claim to ethics expertise is not based on the truth of one's judgments but on one's ability to provide a coherent justification for them” (Yoder 1998, p. 13). In other words, the ethics expert is not the one who knows best which allocative criteria are substantively just, but rather the one who can provide the best reasons and justifications for the chosen criteria. Since, as we argued above, a coherent justification of the chosen allocative criterion is needed, there are good reasons to consider such expertise relevant.

Of course, the ethics expertise is not enough to fill the gap. Allocative criteria do not apply in an abstract setting, but rather within a complex institutional system of laws and rules. To this end, other figures should be involved in the definition of criteria, such as those with legal, economic (Daniels and Sabin 2008, pp. 100–101), and social expertise, patient rights advocates (Daniels and Sabin 2008, p. 172), etc. It is beyond the scope of the paper to provide a detailed list of expertise that should be included in the committee to decide the allocative criteria. Here, we just argue that multifaceted expertise represents a necessary element for ensuring a fair and legitimate allocative decision.

However, although crucial and indispensable in its interdisciplinary articulations, expertise alone is not sufficient. In the next section, we provide reasons supporting this argument and advocating for the inclusion of a political representative in the committee, who should also make the final decision.

## Taking democracy seriously in micro-allocative decisions: the role of the political representative

Thus far, we have argued that, to meet the formal requirements of justice, the attending physician cannot independently determine the allocation criteria. Such a decision risks being arbitrary, non-public, and non-transparent, failing to uphold the formal requirement of equality. Moreover, while potentially capable of providing a public, transparent decision of allocative criteria, the team of doctors may be deficient in offering the same level of transparency regarding the reasons behind such criteria and their appropriate justification, as this choice requires various types of expertise to provide proper justifications, such as ethical, legal, economic, and so on. In this section we argue, however, that although an interdisciplinary team of experts is necessary, it is not sufficient for a fair and legitimate choice. We advocate including a political representative within the committee in charge of deciding the allocation criteria in the micro-allocative context of health care.^6^ In our perspective, the political representative should even be held responsible for the final decision, which means that other experts within the committee should not be able to vote or support a specific and substantive solution.

The political representation within the committee may consist of either a single or multiple representatives. Although we do not extensively argue in favor of a specific option here, we highlight that involving more than one political representative within the committee can have positive effects. For instance, the inclusion of five political representatives reflecting the current political scenario—three from the majority and two from the minority—can enhance accountability and mitigate issues related to the tyranny of the majority by giving a voice to the minority's perspective. More specifically, the discussion among the different allocative options and the decision-making process would gain clarity thanks to the dialectic that emerges between different values and points of view. Despite this brief consideration, within this analysis we will adopt the expression “political representative” to indicate the political component within the committee, whether composed of one or more members.

The thesis that attributes the allocative decision to the politician can be supported by at least two arguments: the democracy argument and the consistency argument.

### The democracy argument

The political representative should be in charge of the final allocative choice because allocative decisions must undergo democratic validation. Urbinati and Warren (2008) note that if one considers, as most democratic theorists do, that individuals are morally and legally equal and equally capable of self-determination, it follows that when citizens are affected by collective decisions, those decisions should include the participation of those who are affected. As long as a national healthcare system exists, the allocation of healthcare resources is a choice that involves all citizens. But this not only requires that the decision on allocative criteria respect the formal requirements of justice discussed earlier, such as the conditions of publicity and transparency, the condition of relevance, and the principle of treating like cases alike. Citizens should also be placed in a position to express their personal preferences on this issue or, as we will argue, at least on the individuals responsible for setting the rules.

Applying the tools of democracy to allocative choices not only provides justification for the voices of those involved to be heard, but also offers a method for resolving disagreements among people involved. Despite almost always intertwined, from a conceptual point of view conflicts or disagreements can be broadly distinguished in disagreements on *facts*—given a common objective x, two different strategies y and z are proposed; citizens disagree regarding the effectiveness of y and z to achieve x—or on *values*—Is the common goal x or w? Although in the allocative scenario we consider both types of disagreement may arise, in this analysis we set aside the former—assuming no disagreement on facts—and focus on the latter, since we want to stress that allocative choices involve values, which are likely to be diverse and conflicting in pluralistic societies. This precise reason creates the need for democratic validation. Otherwise, this choice would not be legitimate. While disagreements on facts can, in principle, be resolved through a method generally shared and deemed reliable by reasonable people—the scientific method—no such method seems to be recognized for disagreements on values, especially when the voices of a large number of people must be considered. For instance, reasonable people can still disagree on issues such as abortion or euthanasia. It follows that value disagreement seems at least harder to solve than disagreement on facts. In light of this hard-to-solve disagreement, the democratic procedure offers a solution, namely the majority rule. By applying the majority rule, the preferences expressed by the population on a specific issue can be observed and incorporated into the final decision. The majority rule can be directly applied, especially to settle profoundly value-based issues. This application has some historical examples.^7^ However, allocative choices, like most political choices, are complex as they involve very specific situations that require an accurate awareness of the context in which such decisions are applied. Therefore, it seems difficult to imagine that citizens could directly express their preferences through consultative tools like referendums when it comes to deciding allocative criteria. It is more plausible, instead, that the preferences, values, and opinions of citizens are represented through the election of political representatives who, in modern democracies, act for the people and stand for them. In other words, in a democratic context, people would grant their representatives the ability to legitimately make choices that are value-laden. Therefore, these representatives will also have the task of making *substantive* decisions regarding allocative criteria in healthcare, necessarily taking into account the will of the people and assuming moral responsibility for the decisions. The political representative will also be responsible for engaging with and informing the public debate on this choice by presenting their arguments in a way that fosters dialogue with public opinion (Urbinati and Warren 2008). This dialogue should not be limited to the time of their election but should continue throughout their political mandate.

In light of these reasons, we can reject the idea that only the team of experts should make the decision, as they would neither be entitled nor legitimized to make any substantive decisions regarding allocative criteria. For a decision on substantive justice to be fair and legitimate in a democratic context, it should be made by a political representative.^8^

Finally, it should be noted that this argument is not necessarily committed to supporting the idea that the political representative must be directly elected by the citizens; they can also be appointed by those who are directly elected. This is a well-established practice in representative democracies like the UK and Italy.^9^ However, the aim of this paper is not to establish the procedure for appointing the political representative, but only to provide reasons to argue that there is a need for some form of democratic legitimation for the person who takes final responsibility for a fair and legitimate decision of allocative criteria.^10^

### The consistency argument

Up to this point, we have limited our reflections to cases of micro-allocation. However, this is only one class of allocative issues. It is possible to identify others, including what is called macro-allocation, which involves deciding how to distribute resources among different sectors—for instance, healthcare, education, etc. Macro-allocative decisions are value-laden and concern substantive justice issues: for example, should we prioritize equal opportunity through education or access to healthcare? Should we promote property rights or greater distribution of healthcare resources? In this case, it is widely accepted that the government of a country, or other representative bodies, such as parliament or local institutions, should make these decisions. However, since micro-allocative decisions are also value-laden, it is unclear why these should not be made by a political representative, while macro-allocative decisions are. Therefore, for reasons of consistency, we should either include a political representative in healthcare micro-allocative decisions or question the role of the government and other representative bodies in deciding on these critical aspects of our lives.

### Constraints for the political representative decisions

A crucial clarification is needed. Stating that the political representative must have the final responsibility for decisions regarding allocative criteria does not imply that their choices will automatically be fair and legitimate, regardless of what those choices are. The political representative’s decision should be constrained by various factors that need to be respected to ensure the legitimacy of the choice. For this reason, while the role of the politician is necessary, it is not sufficient on its own, and their decision must be supported by a team of experts to the extent we discussed in Sect. “Who should decide? Some possible answers”. Primarily, the politician’s choice must adhere to the formal justice requirements. As said, the allocative decision must not only be public and transparent, but also supported by relevant reasons that can be appropriately and coherently justified to citizens. In this context, an ethics expert can provide support in identifying values within different options and in evaluating which decisions can be appropriately justified based on the value commitments of the political representative. The chosen criteria must also be in line with scientific facts and be implementable, meaning that medical experts should assist the politician in making a decision that is operationally feasible and supported by evidence deemed reliable by the scientific community. Finally, the decision must also be consistent with the current legal framework. This requirement ensures that anti-discrimination norms typical of Western democracies, often embedded in constitutions, are respected, as well as existing regulations in the field. Again, despite these significant constraints, there is still room for the politician to make substantive justice choices: for example, there will be multiple viable options that are feasible, supported by justifiable reasons, acceptable to all citizens, and in line with the current legal framework. Within these boundaries, there is room for the substantive choice of the political representative, who, by virtue of the citizens' mandate, is in the best position to make fair and legitimate decisions.

## Two possible critiques

In the previous paragraphs, we have built the case for allowing political representatives to decide the allocative criteria. We acknowledge that our proposal may give rise to various objections. We attempt to address the two that we consider most relevant, namely the too-specificity of the decision critique and the catastrophic outcomes critique. In general terms, these two criticisms can be framed using the distinction proposed by Scharpf (1997), according to which democratic legitimacy is a two-dimensional concept encompassing both the inputs and outputs of the political system. Both dimensions are essential to democracy, understood as a collective effort of self-determination. More specifically, democracy requires mechanisms and procedures that ensure that collective decisions are a direct or indirect reflection of citizens’ preferences (input dimension), while also ensuring that these decisions are effectively implemented, achieving the goals that matter to citizens and avoiding potential risks (output dimension). The two objections we will analyze below suggest that our position secures the input dimension but falls short in ensuring the output dimension.

### The too-specificity of the decision critique

Someone may argue that the technical intricacy characterizing micro-allocative decisions is too predominant to allow a political representative to make an effective judgment; therefore, a politician should not be allowed to engage in these tricky questions, since it may undermine the output dimension of democratic legitimacy due to their lack of skills. We already acknowledged that healthcare micro-allocation is characterized by a very high degree of specificity. These decisions often involve intricate medical details and complex scenarios that require specialized knowledge. However, the values at play can, at a general level, be easily understood even by those who are not medical professionals. Decisions such as prioritizing younger individuals over older ones, prioritizing those who have chosen to vaccinate over those who have not, or deciding to leave these choices to chance are certainly complex but can be comprehended and addressed at a general level without necessarily having highly specialized expertise. With regard to these issues, the politician is required to identify which values should inform the decision, in collaboration with the expert members of the committee, where these values will be discussed and articulated. Here, we are not claiming that technical experts are unnecessary, but, as discussed above, that their expertise is not sufficient. Furthermore, from our perspective, political representatives should be informed and trained by committee experts. Even highly technical decisions in parliaments benefit from the support of state apparatuses to ensure accountability. Moreover, the fact that a political representative joins the committee we envision does not mean that they cannot have prior expertise in sectors related to the micro-allocative decision. For instance, they could be a doctor, understanding the feasibility of allocative decisions, or a lawyer familiar with the regulatory framework. Nevertheless, as a political representative, they possess an independent mandate to take value-laden decisions, ensuring accountability and, ultimately, promoting public debate on the matter.

### The catastrophic outcomes critique

Others may argue that allowing the voice of a politician in this decision could lead to catastrophic, unequal, and discriminatory outcomes. In this sense, the output dimension of democratic legitimacy is undermined, as the outcome may risk compromising fundamental values that people care about, such as the right to non-discrimination. This critique does not do justice to our proposal. While the allocative choice is ultimately made by the political representative, it is informed by the work of experts who ensure that the decision is, as already said, consistent with the current legal framework, supported by sound reasoning, and operationally feasible. Within this framework, it is unlikely to envisage “catastrophic decisions,” defined as undesirable in the democratic context in which we place this reflection. Similarly to how laws promulgated by parliament must align with the constitution and other regulatory frameworks, the allocative choice would be “protected” by expert members of the committee from “catastrophic” outcomes. If the proponents of such a critique continue to consider the outcome of the decision made by the political representative as “catastrophic”, despite it adhering to the requirements and conditions endorsed by experts, they should be prepared to embrace the following, controversial, claims: (a) the democratic method in place in current Western democracies may be inadequate not only in this context but also in other contexts, such as political elections; b) value conflicts could be solved through access to an objective moral truth by a group of experts who would have the task of applying it, regardless of the preferences and values of the other people involved in the decision. Of course, the political representative in practical contexts may choose allocative criteria that are problematic in democratic terms– that is, in contrast with the legal framework, lacking sound reasoning or not operationally feasible–even with the support of the expert board. However, democratic institutions can use revision tools to challenge such a decision and request an adjustment, thus avoiding the catastrophic outcome.

## Conclusion

Structural scarcity in healthcare may prevent everyone from accessing resources, making it necessary to establish allocative criteria. This need has been widely debated in the bioethical field, with specific focus on issues of substantive justice and issues of formal justice related to how substantive criteria are determined. In this paper we explored a relatively negletcted but pivotal question: who should define the allocative criteria? After outlining the formal conditions that make an allocative decision fair and legitimate, we explored several possible answers to this research question, concluding that the criteria should be ultimately determined by a political representative, supported by a board composed of experts from diverse fields. We proposed two arguments to justify our position: the democracy argument, which is the most salient, and the consistency argument. We acknowledged that our position may raise potential criticism, and we preemptively defended it against two major critiques: the too-specificity of the decision critique and the catastrophic outcomes critique.

There are strong reasons to claim that our perspective ensures the fairest and most legitimate decision-making process in the context of micro-allocation. Nevertheless, we are aware that our analysis presents limitations. The crucial role played by experts has been underlined several times and some professionals who should be part of this committee have been listed, such as the ethicist. However, a complete list of all the profiles that should be involved, with related supporting arguments, is missing. Moreover, we did not address whether the political representative should possess specific skills, which go beyond democratic legitimacy, to be responsible for making decisions regarding allocative criteria. Likewise, we did not elucidate the committee's functioning process, nor did we comprehensively detail the procedure through which experts are elected or appointed, and how long they remain in charge. The cited examples represent some of the aspects that have been overlooked. They are undoubtedly relevant, but lie outside the aim of this paper, which mainly focuses on who should define allocation criteria and on what basis. 

More research is needed to explore the unaddressed issues and ensure preparedness in dealing with allocative policies developed at national and international levels. The structural scarcity of resources makes it urgent to strengthen operational readiness for countries to handle complex situations with specific focus on healthcare emergencies in which micro-allocative choices emerge with crucial relevance. The COVID-19 pandemic has demonstrated the effects a lack of preparedness can have for the achievement of fair and legitimate allocation choices. Advanced planning and preparedness are critical to mitigate the risks posed by emergencies and outbreaks, and each country should rely on updated preparedness policies in order to have available an adequate framework in which fair allocation choices can be made.

## Data Availability

Not applicable.

## Footnotes

1 Hereafter we will use the term “allocation” to indicate “micro-allocation”.

2 In this article, we mainly rely on a normative concept of legitimacy, focusing on good reasons and, more broadly, on the justification of the imposition of micro-allocative decisions in a democratic context. This notion differs from the descriptive concept of legitimacy, which pertains to elements such as people’s convictions and beliefs that render an authority’s exercise of power acceptable to them and foster a willingness to obey (Peter 2023; Weber 1964). Notwithstanding, we acknowledge that a normative discussion of legitimacy alone is not sufficient for an all-things-considered evaluation of the procedures and actors involved in decisions regarding the allocation of scarce resources, and that considerations related to the empirical dimension are also relevant. For instance, the effectiveness and proper implementation of an allocative choice can be influenced by the extent to which those involved factually recognize the necessity of rationing and the related decisions. We thank an anonymous reviewer for raising the point about the descriptive form of legitimacy.

3 We primarily use the pronouns ‘they/them’ to refer to individuals, but we use ‘she/her’ when there is a potential for confusion.

4 For a comprehensive overview of the critiques on such approach, see Battisti (2023).

5 For further discussions on the argument that expert participation can be seen as a means of increasing transparency and information, see Fischer (2009, 2003) and Landwehr (2009).

6 This proposal may share some similarities with others in different fields. See Leclerc and Herrera (2000).

7 It was May 17, 1981, when Italian citizens were called to express their opinions on five referendum questions. One of these proposed to almost completely repeal law no. 194 on voluntary termination of pregnancy and 68% of voters rejected the proposal.

8 Our argument is not committed to claiming that political representatives should always be directly involved in all decisions pertaining collective issues. These figures should be involved as long as it remains an open question concerning which substantive values should guide the decision. Conversely, when the values and interests to be promoted are clearly and definitely established (e.g., when a law has already been promulgated and has resolved the substantive debate), it can be is reasonable for experts to make decisions without the need for political representatives.

9 In the UK, citizens cannot vote for the new Prime Minister. They can only vote to elect the local MP that will represent a local area in the House of Common. The political party that wins the most seats in the House of Commons at a general election usually forms the new government. Its leader becomes Prime Minister and is appointed by the Monarch. Also in Italy, which is a parliamentary republic, the Prime Minister is appointed by the President of the Republic who, in turn, is elected by the Parliament, which is the body directly elected by the people.

10 We believe that this argument can at least help mitigate the problem highlighted by Lauridsen and Lippert-Rasmussen (2009), namely that we cannot identify the reasons fair-minded people will consider relevant without some democratic debate. Nevertheless, further reflection is needed to appropriately address such a criticism; we simply note here that including a political representative in the decision-making process could be a promising strategy to resolve some of the issues that the relevance condition may face.
